# Case of post‐orgasmic illness syndrome associated with hypogonadism

**DOI:** 10.1002/iju5.12184

**Published:** 2020-07-01

**Authors:** Teppei Takeshima, Shinnosuke Kuroda, Yasushi Yumura

**Affiliations:** ^1^ Department of Urology, Reproduction Center Yokohama City University Medical Center Yokohama Japan

**Keywords:** ejaculatory disorder, hypogonadism, post‐orgasmic illness syndrome, testosterone replacement therapy

## Abstract

**Introduction:**

Post‐orgasmic illness syndrome is a rare condition that occurs after ejaculation and persists for 2–7 days and is characterized by flu‐like symptoms, which can significantly reduce quality of life.

**Case presentation:**

A 21‐year‐old unmarried man was referred to our hospital due to flu‐like symptoms that developed after ejaculation by masturbation and persisted for about 2 days. The patient’s free testosterone level was slightly lower than normal. Nonsteroidal anti‐inflammatory drugs were initially administered and helped relieve headache and muscle pain. Thereafter, the patient was able to ejaculate three times a week. In addition, after administering testosterone enanthate once or twice a month, his general fatigue significantly improved, and he could ejaculate every day.

**Conclusion:**

The pathophysiology of post‐orgasmic illness syndrome has not been fully elucidated. The treatments for this condition must be accurately selected according to pathophysiology.

**Abbreviations & Acronyms**
AMS: aging male symptom
hCG: human chorionic gonadotropin
NSAIDs: nonsteroidal anti‐inflammatory drugs
POIS: post‐orgasmic illness syndrome
QOL: quality of life
SHIM: sexual health inventory for men
SPT: skin pick test
TRT: testosterone replacement therapy

**Keynote message**

POIS is a rare condition that occurs after ejaculation and persists for 2–7 days and is characterized by flu‐like symptoms. We experienced a case of POIS associated with hypogonadism. We had successfully treated symptoms of POIS with administration of NSAIDs followed by TRT.

## Introduction

POIS is a rare condition that occurs after ejaculation and persists for 2–7 days and is characterized by flu‐like symptoms.^1^ In 2001, Waldinger and Schweitzer first reported about POIS,^2^ and proposed five preliminary diagnostic criteria for POIS, as shown in Table 1.^1^, ^3^ The symptoms of POIS were classified into seven clusters, which can impair a patient’s motivation to ejaculate and QOL. However, the actual pathophysiologies of POIS have not been fully elucidated; therefore, effective treatments have not been established thus far. They also have reported about two patients treated with subcutaneous immunotherapy with autologous semen, which decreased the symptoms.^4^ However, randomized controlled trials of immunotherapy have not been performed; thus, the efficacy of this treatment has not been validated. By contrast, Ashby *et al*. have hypothesized that the production of inflammatory cytokines is the mechanism associated with POIS and that the administration of NSAIDs is effective.^5^ Herein, we report a case of POIS associated with hypogonadism that was successfully treated with TRT combined with NSAIDs.

**Table 1:** Five preliminary diagnostic criteria for POIS

Criterion	Description
1	≧1 of the following symptoms: sensation of flu‐like state, extreme fatigue or exhaustion, weakness of musculature, experiences of feverishness or perspiration, mood disturbances and/or irritability, memory difficulties, concentration problems, incoherent speech, congestion of nose, or watery nose, itchy eyes
2	All symptoms occur immediately (e.g. seconds), soon (e.g. minutes), or within a few hours after ejaculation that is initiated by coitus and/or masturbation and/or spontaneously (e.g. during sleep)
3	Symptoms occur always or nearly always (i.e. >90% of ejaculation events)
4	Most of these symptoms last for 2−7 days
5	Symptoms disappear spontaneously

## Case presentation

A 21‐year‐old unmarried man was referred to the men’s health clinic of our hospital due to flu‐like symptoms that developed after ejaculation by masturbation and that persisted for about 2 days. He had his first masturbation with hand thrust at the age of 19 years. Since then, he experienced general fatigue, chills, headache, nasal congestion, and muscle pain almost every after ejaculation. The symptoms lasted for about 2 days and spontaneously disappeared. Moreover, the frequency of his morning erection significantly decreased. The patient’s symptoms met the POIS criteria; therefore, he was diagnosed with POIS.

The growth of his pubic hair and penis was normal (each with a Tanner’s grade of 5). On physical examination, his testicular volume was a little atrophic on the left side (12 mL).

His total testosterone level (4.75 ng/mL), gonadotropic level (luteinizing hormone: 3.5 mIU/mL and follicle stimulating hormone: 8.7 mIU/mL) was normal, and free testosterone level (10.3 pg/mL) was mildly low.

The patient’s AMS score was 45 (psychological: 10, somatic: 19, and sexual: 16), and his SHIM score was 3.

Antihistamine drugs were administered for allergic symptoms. However, the treatment was not effective. For headache and muscle pain, celecoxib 200 mg, which is an NSAID, was administered daily just after ejaculation. Immediately after the intake of the drug, headache and muscle pain were relieved, and the patient was able to ejaculate 3 days per week. However, general fatigue did not improve.

Thereafter, in addition to NSAIDs, 250 mg of testosterone enanthate was administered as a TRT every 2 weeks because the patient’s serum free testosterone level was lower than 70% of the average value in young adult men.^6^ His general fatigue significantly improved, and morning erection has been achieved every day. Therefore, he can ejaculate everyday by masturbation. The patient’s AMS score decreased to 21 and SHIM score increased to 7. The interval of drug administration was changed from 2 to 4 weeks. However, no recurrence of symptoms was observed.

TRT was switched to testosterone ointment (Glowmin^®^; Daito Pharma, Tokyo, Japan), and his symptoms continually improved.

## Discussion

POIS is a rare condition that occurs after ejaculation and persists for 2–7 days and is characterized by flu‐like symptoms. Waldinger *et al*. have proposed five preliminary diagnostic criteria for POIS, as shown in Table 1.^1^ POIS is classified in two types: primary type (from the first ejaculation during puberty or adolescence) and secondary type (from ejaculation later in life).^7^ POIS develops within 30 min after ejaculation in 87% of men and persists for an average of 4.6 ± 2.8 days. Moreover, the average frequency of sexual intercourse in 73% of patients is 1.04 ± 1.00 times per week, and 17.8% engage in sexual intercourse once in 2–6 months. However, 6.7% of patients abstain from intercourse.^7^ Therefore, this syndrome can be a psychological burden on patients, which can lead to decreased ejaculation frequency, avoidance of sexual activities and romantic relationships, schedule problems, and struggles in preventing eroticism.^1^ Therefore, POIS could significantly reduce a patient’s QOL.

However, the pathophysiology of POIS has not been fully elucidated. Waldinger *et al.* hypothesized that POIS is an autoimmune or allergic disorder caused by an inflammatory response of the urethral mucosal epithelium to antigens in a patient’s own seminal fluid. A total of 33 patients with POIS underwent SPT; 88% had a positive reaction to their own semen.^3^ Two patients had hyposensitization therapy with autologous semen, and both were successfully treated, with 60% and 90% improvement in POIS complaints at 31 and 15 months.^3^ However, the efficacy of this therapy was not assessed in a randomized placebo‐controlled study. Alternatively, Ashby and Goldmeier hypothesized that POIS is caused by a disorder in the cytokine and neuroendocrine response.^4^ In this study, the administration of NSAIDs (diclofenac) was effective in alleviating symptoms (up to 80% improvement), and the patient experienced increased sexual frequency from 2 to 4 times a month.^4^ In our case, the patient’s non‐specific IgE level did not increase, as previously reported,^8^ and SPT was not performed. Alternatively, NSAID was administered and had a partial efficacy.

Most studies about the physiological involvement of testosterone in an ejaculatory disorder used animal‐based models,^9^ and human‐based studies of the relationship between serum testosterone level and ejaculatory function are extremely limited. Several studies have reported that ejaculatory disorders during sexual intercourse are associated with decreased serum testosterone levels.^10^ However, few previous studies has shown that POIS is associated with testosterone.^11^ In our case, though decline of his free testosterone level was modest, POIS associated with hypogonadism was suspected. Because the patient in our case was not married and did not have any desire to have babies, he chose TRT once or twice a month though we proposed self‐injection of hCG to avoid possible testicular atrophy due to the negative feedback of gonadotropin. TRT was markedly effective. After switching the treatment to testosterone ointment, his symptoms continually improved. Bolanos and Morgentaler also reported a case of POIS associated with hypogonadism, which was successfully treated with administration of hCG.^11^ Herein, we report a case of POIS associated with hypogonadism, which was different from allergy‐associated condition, thereby treatments must be accurately selected according to pathophysiology.

## Conflict of interest

The authors declare no conflict of interest.
